# Synthetic Oxoisoaporphine Alkaloids: In Vitro, In Vivo and In Silico Assessment of Antileishmanial Activities

**DOI:** 10.1371/journal.pone.0077560

**Published:** 2013-10-29

**Authors:** Eduardo Sobarzo-Sánchez, Pablo Bilbao-Ramos, Maria Dea-Ayuela, Humberto González-Díaz, Matilde Yañez, Eugenio Uriarte, Lourdes Santana, Victoria Martínez-Sernández, Francisco Bolás-Fernández, Florencio M. Ubeira

**Affiliations:** 1 Departamento de Tecnología Farmacéutica, Facultad de Farmacia, Universidad de Santiago de Compostela, Santiago de Compostela, Spain; 2 Facultad de Ciencias de la Salud, Universidad Autónoma de Chile, Santiago, Chile; 3 Departamento de Parasitología, Facultad de Farmacia, Universidad Complutense de Madrid, Madrid, Spain; 4 Departamento de Farmacia, Universidad Cardenal Herrera, Valencia, Spain; 5 Departamento de Microbiología y Parasitología, Facultad de Farmacia, Universidad de Santiago de Compostela, Santiago de Compostela, Spain; 6 Departamento de Farmacología, Facultad de Farmacia, Universidad de Santiago de Compostela, Santiago de Compostela, Spain; 7 Departamento de Química Orgánica, Facultad de Farmacia, Universidad de Santiago de Compostela, Santiago de Compostela, Spain; The Ohio State University, United States of America

## Abstract

Leishmaniasis is a growing health problem worldwide. As there are certain drawbacks with the drugs currently used to treat human leishmaniasis and resistance to these drugs is emerging, there is a need to develop novel antileishmanial compounds, among which isoquinoline alkaloids are promising candidates. In this study, 18 novel oxoisoaporphine derivatives were synthesized and their possible antileishmanial activity was evaluated. The in vitro activity of these derivatives against *Leishmania amazonensis* axenic amastigotes was first evaluated, and the selected compounds were then tested in an inhibition assay with promastigotes of *L. infantum*, *L. braziliensis*, *L. amazonensis* and *L. guyanensis,* and with intracellular amastigotes of *L. infantum* and *L. amazonensis*. Finally, the most active compounds, OXO **1** (2,3-dihydro-7*H*-dibenzo[*de,h*]quinolin-7-one) and OXO **13** (2,3,8,9,10,11-hexahydro-7*H*-dibenzo[*de,h*]quinolin-7-one), were tested in BALB/c mice infected with *L. infantum*. Treatment of mice at a dose of 10 mg/kg with OXO **1** yielded significant reductions (p<0.05) in parasite burden in liver and spleen (99% and 78%, respectively) whereas with OXO **13** were not significant. Although previous reports suggest that this family of molecules displays inhibitory activity against monoamine oxidase A and acetylcholinesterase, these enzymes were not confirmed as targets for antileishmanial activity on the basis of the present results. However, after development of a new bioinformatics model to analyze the *Leishmania* proteome, we were able to identify other putative targets for these molecules. The most promising candidates were four proteins: two putative pteridine reductase 2 (1MXF and 1MXH), one *N-*myristoyltransferase (2WUU) and one type I topoisomerase (2B9S).

## Introduction

Leishmaniasis is a vector-borne disease caused by an obligate intra-macrophage protozoan parasite. The disease, which is endemic in large areas of tropical and subtropical countries, is caused by more than 20 species of *Leishmania* and transmitted to humans by more than 30 different species of phlebotomine sandflies. The clinical manifestations of leishmaniasis largely depend on complex interactions between the virulence of the infecting *Leishmania* species and the immune responses of the host. The three major clinical syndromes that are recognized in human disease are visceral, cutaneous and muco-cutaneous leishmaniasis [1].

Leishmaniasis is a major public health problem and the burden is increasing. An estimated 2 million new cases (1.5 million cases of cutaneous leishmaniasis and 500,000 of visceral leishmaniasis) occur annually, and about 12 million people are currently infected [2]. This epidemiological scenario has worsened because dogs are the principal reservoirs as well as suffers of the disease, for which fully successful treatment is still lacking [3].

At present, the following drugs are used to treat human leishmaniasis: pentavalent antimonials, paromomycin, amphotericin, miltefosine and pentamidine. However, there is an urgent need to develop new antileishmanial drug candidates to overcome problems such as toxic side effects, route of administration, long-term treatment and the generation of resistance mechanisms to current drugs [4].

Possible new antileishmanial compounds include oxoaporphines, which are widely distributed in nature and are of pharmacological importance in the treatment of certain cancers and parasitic diseases. For example, liriodenine is a oxoaporphine isolated from a variety of species belonging to the families Annonaceae, Magnoliaceae, Menispermaceae, Monimiaceae and Rutaceae [5], and which has been shown to be active against *L. amazonensis*, *L. braziliensis*, *L. donovani*, *L. guyanensis* and *L. major* [6], [7], [8], [9]. Liriodenine is a potent inhibitor of topoisomerase II and also displays other types of activity, such as antibacterial, antifungal, antitumor and antiviral activity [5], [10]. Other oxoaporphines reported to possess antileishmanial activity include dicentrinone, obtained from the stem bark of *Duguetia furfuracea*, and *N*-methylliriodendronine, isolated from *Stephania dinklagei*; these have been shown to be active against *L. braziliensis* [10] and *L. donovani* [7], respectively. In addition to oxoaporphines, a small group of isomers (oxoisoaporphines) that occur in some Chinese medicinal plants, such as the roots of *Menispermum dauricum*, may exert leishmanicidal activity. However, these isomers have not been well studied, mainly because of the low concentrations at which they occur in the plants. Given the potential interest in these compounds for treating leishmaniasis, the aim of the present study was to investigate, for the first time, the in vitro and in vivo leishmanicidal activity of several novel synthetic oxoisoaporphine compounds. In addition, we have also developed a new bioinformatics tool for predicting putative targets of these molecules in the parasite proteome.

## Materials and Methods

### Ethics Statement

All procedures for animal manipulations were approved by the Institutional Animal Care and Use Committee of the Complutense University of Madrid, following Spanish law.

### Synthesis of Oxoisoaporphine Derivatives (OXO 1–18)

The compounds used in the present study were synthesized according to a previously described general procedure [11]–[13].

### Reference Drugs

Miltefosine and rasagiline were purchased from Sigma-Aldrich (Madrid, Spain).

### In vitro Assays with *Leishmania* Axenic Amastigotes

For preliminary screening of potential active compounds, axenic amastigotes of *L. amazonensis* (strain MHOM/BR/76/LTB-012) were arbitrarily chosen and cultured according to Estevez et al. [14]. The axenic amastigotes were cultured in medium supplemented with 20% foetal bovine serum (FBS) (Sigma-Aldrich) and incubated at 32°C with 5% CO_2_ in 25 cm^2^ culture bottles. In order to determine the activity of the compounds, a colorimetric method with 3-(4,5-dimethylthiazol-2-yl)-2,5-diphenyltetrazolium bromide (MTT; Sigma-Aldrich) was used, as previously described [15]. The compounds were dissolved in dimethylsulfoxide (DMSO) and added to wells of microtiter plates containing 100 µl of cultivated amastigotes (in logarithmic growth phase) to final concentrations ranging from 0.05 µg/ml to 50 µg/ml. The plates were incubated for 72 h, and 10 µl of the MTT solution (10 mg/ml in PBS) were then added to each well and incubated for a further 4 h. The reaction was stopped by addition of 100 µl of isopropanol-sodium dodecyl sulphate (SDS). Finally, the optical density (OD) was read at 570 nm. All experiments were carried out in triplicate.

### In vitro Assays with *Leishmania* Promastigotes

For these assays, the following *Leishmania* species were used: an autochthonous isolate of *L. infantum* (MCAN/ES/92/BCN83) obtained from an asymptomatic dog in the Priorat region of Catalunya (Spain) and kindly donated by Prof. Montserrat Portús (University of Barcelona); and *L. braziliensis* 2903, *L. amazonensis* (MHOM/Br/79/Maria) and *L. guyanensis* 141/93, kindly provided by Prof. Alfredo Toraño (Instituto del Salud Carlos III, Madrid). The promastigotes were cultured at 26°C in Schneider’s Insect Medium (Sigma-Aldrich) supplemented with 10% heat-inactivated FBS and 100 U/ml of penicillin plus 100 µg/ml of streptomycin (Sigma-Aldrich), in 25 ml culture flasks.

To test the oxoisoaporphine compounds, promastigotes of each species (2.5×10^5^ parasites/well) were cultured in 96-well microtiter plates. The compounds were dissolved in DMSO and diluted in the culture medium at concentrations ranging from 0.8 to 100 µg/ml in a final volume of 200 µl. Miltefosine was used as the reference drug. After incubation for 48 h at 26°C, 20 µl of 2.5 mM resazurin (Sigma–Aldrich) solution were added to each well, and the fluorescence intensity (535 nm -excitation wavelength- and 590 nm -emission wavelength) was measured in a fluorometer (Infinite 200, Tecan Group Ltd, Männedorf, Switzerland). All tests were carried out in triplicate.

### In vitro Assays with *Leishmania* Intracellular Amastigotes

The fluorometric assay for drug screening against intracellular amastigotes was carried out as described by Bilbao-Ramos et al. [16]. Briefly, 5×10^4^ macrophages and stationary promastigotes at a 1∶10 ratio in 200 μl/well of culture medium were seeded and the plates were incubated for 24 h at 33°C, 5% CO_2_ in humidity chamber. The temperature was then increased to 37°C for another 24 h. The cells were then washed several times to remove free non-infective promastigotes; the final washing medium was replaced with 200 μl/well of culture medium containing 2-fold serial dilutions of the test compounds in a triplicate assay. Miltefosine was included as reference drug. After incubation of the plates for 48 h at 37°C, 5% CO_2_, the culture medium was replaced with an equal volume of the lysis solution (RPMI-1640 with 0.048% HEPES and 0.006% SDS) and maintained at room temperature for 20 min. The lysis solution was then replaced with Schneider’s medium followed by incubation at 26°C for another 3 days to allow transformation of viable amastigotes into promastigotes and subsequent proliferation. Aliquots of 20 μl of 2.5 mM resazurin were added to each well and the plates were incubated for 3 h. Finally, fluorescence emission was measured as described above.

### Macrophage Cytotoxicity Assays

Toxicity cell assays were carried out as described elsewhere [17]. To test the oxoisoaporphine compounds, the J774.2 cells (EACC 80011428) were seeded (5×10^4^ cells/well) in 96-well flat bottomed microplates and allowed to adhere for 24 h at 37°C in 5% CO_2_. The medium was then replaced with different concentrations of the test compounds, followed by incubation for another 24 h. Each concentration was assayed three times and growth controls were also included. Thereafter, 20 µl of a 2.5 mM resazurin solution were added to each well and the plates were incubated for 3 h. The fluorescence emission was measured as indicated above.

### In vivo Assays

#### Experimental infection

The experimental infections were carried out in groups of 6–8 BALB/c mice, aged 6–8 weeks. Amastigotes of *L. infantum* (MCAN/ES/92/BCN83) were harvested from spleens of infected hamsters and then cultured in NNN medium containing penicillin (200 IU) and streptomycin (2 mg/ml) for 2 days, until transformation into promastigotes. Promastigotes were grown for 7 days, as described elsewhere [18], and were then harvested, washed and resuspended for counting. The animals were anaesthetized with sodium pentobarbital and then infected with 10^7^ promastigotes per animal, administered via the intracardiac route.

#### Treatment

The treatments began on day 35 post-infection and were administered on 3 continuous days. The compounds were first dissolved in DMSO and diluted in physiological saline to provide doses of 2.5, 5 and 10 mg/kg, which were administered daily by the intraperitoneal route in a final volume of 0.1 ml. One group administered with the vehicle alone was used as an untreated control and another group was treated with miltefosine as reference drug. Miltefosine was administered intraperitoneally for purposes of comparison with test compounds. Five days later, the mice were killed and the parasitic burden was estimated.

#### Estimation of parasite burden

The liver and spleen were removed from each animal and homogenised. After eliminating cell debris, the suspension was centrifuged, the supernatants were discarded and the pellets were resuspended as previously described [18]. Aliquots (200 µl) of the suspension were transferred to each well of 96-well microtiter plates containing NNN medium supplemented with antibiotics. The parasite burden was estimated by the limit dilution assay, according to Hill et al. [19] and Titus et al. [20].

### Statistical Analysis

For in vitro assays, the antiparasitic activity and cytotoxic effect of compounds, expressed as IC_50_ (or IC_90_) and CC_50_, respectively, were assessed by multinomial probit analysis. For in vivo assays, the data were analyzed by Tukey’s HSD post-hoc test. Differences were considered significant at *p*<0.05. SPSS v20.0 and Microsoft Excel 2007 software were used for all analyses.

### Determination of the Inhibitory Activity Against the Monoamine Oxidase (MAO) A and B Enzymes

The potential effects of the tested drugs on hMAO activity were previously investigated [21] using the Amplex® Red MAO assay kit (Molecular Probes, Inc., Eugene, Oregon, USA) and microsomal MAO isoforms prepared from insect cells (BTI-TN-5B1-4) infected with recombinant baculovirus containing cDNA inserts for hMAO-A or hMAO-B [22].

### Determination of Inhibition of the Cholinesterase Enzyme

The cholinesterase assay method of Ellman [23] was used to determine the in vitro cholinesterase activity. The assay medium contained 50 mM PBS, pH 8.0, 20 mM dithiobisnitrobenzoate (DTNB; Sigma-Aldrich), 0.165 U/ml of recombinant acetylcholinesterase (AChE) expressed in HEK 293 cells (Sigma-Aldrich) and 0.75 µM substrate (acetylthiocholine iodide; Sigma-Aldrich). The activity was determined by measuring the increase in absorbance at 412 nm in a FLUOstar Optima microplate reader (BMG Labtech GmbH, Offenburg, Germany), at 1 min intervals, for 10 min at 37°C. In dose-dependent inhibition studies, the substrate was added to the assay medium containing enzyme, buffer, and DTNB with inhibitor after incubation for 10 min. All experiments were carried out in duplicate and the results were expressed as means ± SEM. The relative activity is expressed as the percentage ratio of enzyme activity in the absence of inhibitor.

### Bioinformatics Analysis

The model was constructed with protein data obtained from the Protein Data Bank (PDB) [24]. All 128 *Leishmania* Proteins with known ligands (LPs), including both single proteins and protein complexes, were downloaded. Separation of the protein complexes yielded 624 single LPs in total (protein chains). The option known as ligand report was used to collect all Ligand-LP Interactions pairs (LLPIs) reported in the PDB. Only organic drugs and metabolites were considered. The final dataset contains 563 LLPIs, including 122 different ligands and 3,823 non-interacting Ligand-LPs pairs (nLLPIs). The nLLPIs are pairs formed between the 122 ligands and LPs that do not interact with them. The MARCH-INSIDE (MI) software was then used to calculate the values of spectral moments *π_k_ (L_m_*) and *π_k_(P_n_)* for both ligands and LPs sequences, respectively [25]. These molecular descriptors were used as inputs to search for a linear model. The Linear Discriminant Analysis (LDA) method, implemented in STATISTICA version 6.0 (ST) [26], was used to develop a simple linear classifier with the following general formula:
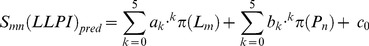
(1)


**Figure 1:**
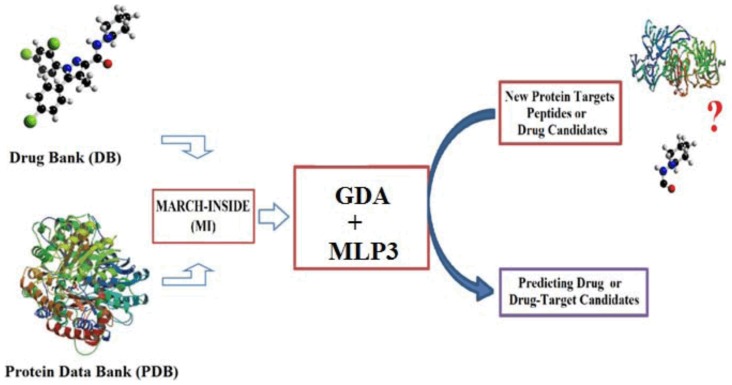
Diagram of the workflow used to develop and use the predictive method.

This equation has only two additive terms; however difference or interaction terms can also be used in non-linear analysis. The model deals with the classification of ligands into two sub-sets: with or without affinity against many different proteins expressed in the proteome of *Leishmania* sp. A dummy variable Ligand-*Leishmania* Protein Interaction (*LLPI_mn_*) was used as input to codify the affinity. This variable indicates either high (*LLPI_mn_* = 1) or low (*LLPI_mn_* = 0) affinity of a given ligand *L_m_* by different LPs *P_n_.* The predicted score *S_mn_(LLPI)_pred_* is the output and it is a continuous dimensionless score that classifies ligands from low to high affinity to the protein target. In the model, a_k_, b_k_, and c_0_ represent the coefficients of the classification function. The statistical significance of the model was determined by calculating the p-value (p) with the Chi-square test. The specificity, sensitivity and total accuracy were also checked to determine the quality-of-fit to data in training. The canonical regression coefficient Rc was used to determine the strength of the linear correlation between the independent inputs (*π_k_*) and dependent variable *LLPI_mn_*. The model validation was corroborated with external prediction series. A diagram of the general workflow used to develop and apply this and other models is shown in Figure 1.

## Results

### In vitro Assays

**Figure 2:**
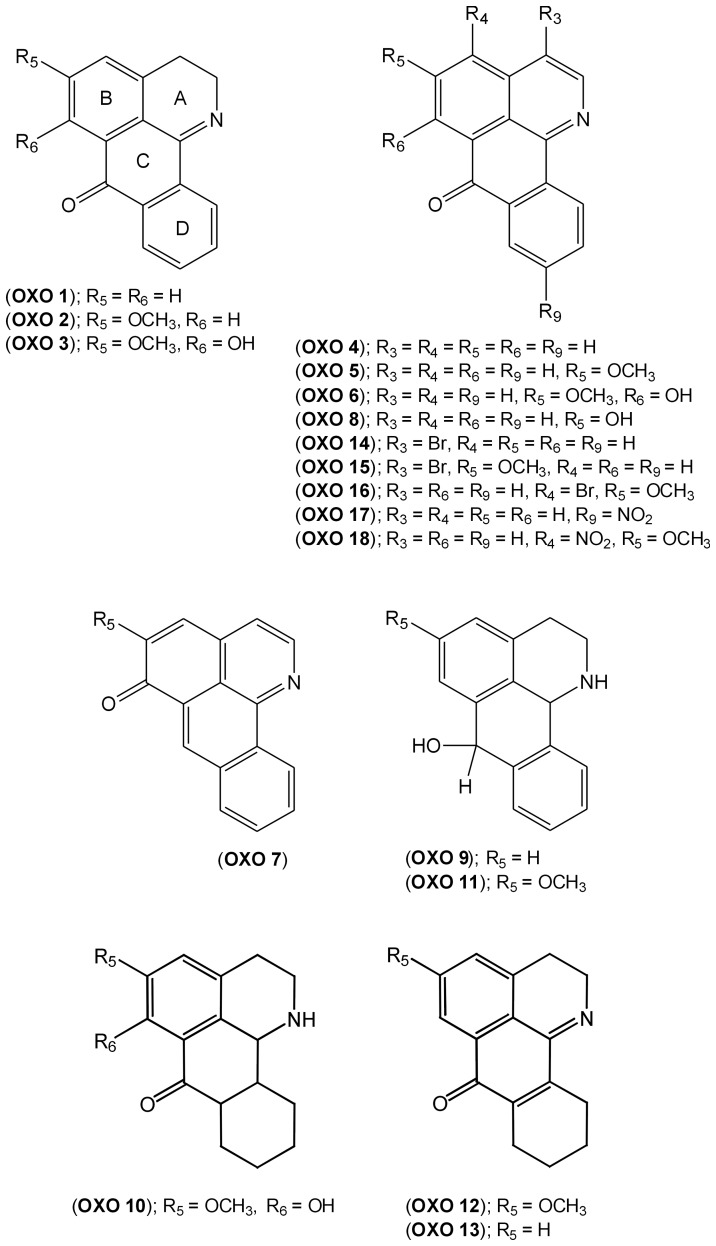
Chemical structures of oxoisoaporphine derivatives (OXO 1–18).

Eighteen different oxoisoaporphine derivatives, OXO **1–18** (Figure 2), were evaluated in vitro in axenic cultures of amastigotes of *L. amazonensis*, to test for possible antileishmanial activity. The inhibitory activity of these compounds was tested at several concentrations ranging from 0.05–50 µg/ml. The results showed that only OXO **1** and OXO **13** rendered maximal inhibition at the lowest concentration of 0.05 µg/ml (Table 1). These pharmacological results appear to indicate that although all tested compounds are chemically related structures, the presence of either a certain type of carbon framework or moieties are important for the in vitro anti-*Leishmania* activity of oxoisoaporphines.

**Table 1:** In vitro assays of axenic amastigotes from *Leishmania amazonensis* with oxoisoaporphine derivatives 1–18.

	Inhibition (%) at concentrations tested				Leishmanicidal activity	
Compound	50 µg/ml	5 µg/ml	0.5 µg/ml	0.05 µg/ml	IC_90_	IC_50_
**OXO 1**	107.8	107.4	105.7	100.6	<0.05	<0.05
**OXO 2**	93.9	84.8	0	0	16.4	0.2
**OXO 3**	98.1	70.8	0	2.00	19.5	5.5
**OXO 4**	97.5	20.3	10.1	2.3	31.1	4.9
**OXO 5**	65.5	0	1.0	3.6	134.6	40.9
**OXO 6**	100.0	88.8	15.2	7.9	9.7	1.5
**OXO 7**	103.4	94.7	58.6	10.3	3.6	0.34
**OXO 8**	98.9	87.4	5.4	3.0	12.8	2.6
**OXO 9**	98.0	7.3	15.9	3.9	31.1	13.3
**OXO 10**	101.5	23.8	10.7	2.8	19.5	3.8
**OXO 11**	100.8	0.1	1.7	1.0	29.438	15.2
**OXO 12**	101.1	101.8	14.1	8.5	2.3	0.5
**OXO 13**	106.9	103.2	108.0	106.7	<0.025	<0.025
**OXO 14**	95.2	0	0	2.0	19.9	10.4
**OXO 15**	95.7	0	2.3	0	19.8	10.8
**OXO 16**	88.0	0	4.1	1.7	27.0	13.3
**OXO 17**	98.3	0	0.5	0.5	18.2	10.6
**OXO 18**	103.3	99.4	0	0	1.6	0.8

IC_90_: 90% inhibitory concentration, IC_50_: 50% inhibitory concentration.

Compounds OXO **1** and OXO **13** were then selected to determine the in vitro effectiveness against promastigotes of four species of *Leishmania* representative of the main clinical forms of the disease: *L. infantum* (as a model of visceral leishmaniasis) and *L. amazonensis*, *L. braziliensis*, and *L. guyanensi*s (as models of cutaneous or mucocutaneous forms). The cytotoxicity of both molecules against the J774.2 macrophage cell line was also tested. Both compounds displayed antileishmanial activity against the 4 species tested with the highest activity recorded against *L. guyanensis* (Table 2). Comparatively, compound OXO **13** was the most active (IC_50_ lower than that recorded for the reference compound, miltefosine), but this compound also displayed a high degree of toxicity (CC_50_ = 31.4 µg/ml compared with 55.4 µg/ml for miltefosine). By contrast, compound OXO **1** showed an intermediate level of activity against all *Leishmania* species, and it was not toxic for the J774.2 cell line. In order to simulate natural physiological conditions in the vertebrate host, the selected compounds were tested against intracellular amastigotes in a newly developed fluorometric assay using *L. infantum* and *L. amazonensis*. Both compounds displayed significant antileishmanial activity, relative to that of the reference drug, against both species. OXO **13** was most active, almost 5 times as active as miltefosine (IC_50_ 4.49±0.64 μg/ml and 4.83±0.61 μg/ml versus 20.9±1.47 μg/ml and 23.7±1.78 μg/ml against *L. amazonensis* and *L. infantum*, respectively) (Table 3). Both products also exhibited good selectivity indexes in comparison with the reference drug.

**Table 2:** In vitro antileishmanial activity against promastigotes and cytotoxic activity of oxoisoaporphine derivatives (OXO 1 and 13).

	IC_50_ (µg/ml)				CC_50_ (µg/ml)
Compound	*L. infantum*	*L. amazonensis*	*L. braziliensis*	*L. guyanensis*	*J774.2*
**OXO 1**	42.1±3.5	31.8±2.2	48.3±2.7	16.3±1.4	>100
**OXO 13**	4.4±0.2	3.6±0.3	4.4±0.2	1.8±0.1	31.4±3.2
**Miltefosine**	7.2±0.6	12.5±0.4	7.2±0.2	7.9±0.5	55.4±4.2

IC_50_: 50% inhibitory concentration; CC_50_: 50% cytotoxic concentration.

**Table 3:** In vitro activity of selected compounds on intracellular amastigotes of *L. amazonensis* and *L. infantum*.

	IC_50_ (μg/ml)		Selectivity Index^a^	
Compound	*L. amazonensis*	*L. infantum*	*L. amazonensis*	*L. infantum*
**OXO 1**	20.6±1.2	56.4±3.8	>6	>3
**OXO 13**	4.5±0.6	4.83±0.6	6.99	6.5
**Miltefosine**	20.9±1.5	23.7±1.8	2.65	2.3

IC_50_: 50% inhibitory concentration.

a Selectivity index: ratio between CC_50_ (as recorded in Table 2) and IC_50._

### In vivo Assays

Finally, both in vitro active compounds were tested in vivo in BALB/c mice infected with *L. infantum*. The results, expressed as mean values (number of parasites × 10^6^) ± standard deviation (SD) of individual data, as assessed by limit dilution assay taken from two independent experiments, are shown in Table 4. Compound OXO **13** was not effective, as the percentage reduction of *Leishmania* amastigotes in liver and spleen did not differ from that obtained for the untreated control. In contrast, compound OXO **1** caused a significant reduction in the parasite burden in spleen and livers of treated mice (Table 4). The reduction of parasite with OXO **1** in livers reached 99±2% (p<0.05) for a dose of 10 mg/kg, whereas the reduction for the same dose in spleens was slightly lower 78±33% but also significant (p<0.05). Considering the toxicity, relative to the reference drug miltefosine, which caused 12.5% mortality at a dose of 5 mg/kg, OXO **1** was not toxic, even at the maximal dose of 10 mg/kg.

**Table 4:** In vivo antileishmanial activity of compounds OXO 1 and OXO 13.

		Liver		Spleen	
Compound	Dose(mg/kg)	Mean	%Red	Mean	%Red
**Control**	–	29.5±8.5	–	466.8±105.1	–
**OXO 1**	2.5	3.3±7.0*	89±24	210.2±219.4	55±47
**OXO 1**	5	9.0±15.1	70±51	249.8±182.1	47±39
**OXO 1**	10	0.4±0.6*	99±2	103.0±154.0*	78±33
**OXO 13** a	2.5	24.3±12.4	18±42	456.5±228.7	2±49
**OXO 13** a	5	25.2±17.9	15±61	402.4±186.7	14±40
**OXO 13**	10	18.3±13.3	38±45	422.0±154.0	10±33
**Miltefosine**	2.5	20.5±14.2	31±48	200.7±224.1	57±48
**Miltefosine**	5†	14.7±11.5	50±39	230.3±186.7	51±40

Reduction in parasite burden in spleens and livers of treated mice (8 animals/group), relative to untreated controls.

a Six animals/group.

† 12.5% mortality.

* *p*<0.05.

### Binding of Oxoisoaporphine Compounds to MAO and AChE Enzymes

To investigate possible enzyme targets that may explain the different capacity of oxoisoaporphine compounds to inhibit the growth of *Leishmania* parasites, we compared the ability of compounds OXO **1** and OXO **13** to inhibit the MAO and AChE enzymes. The enzymes were chosen on the basis of the results of previous studies, which showed that several molecules chemically related to oxoisoaporphines display inhibitory activity against AChE and MAO-A [21], [27], [28]. The data in Table 5 show that both compounds were good inhibitors of MAO-A (data taken from Prado-Prado et al. [21]) and display a significant degree of inhibitory activity against AChE; however, there were no differences in the inhibitory activity of these compounds against MAO-A or AChE enzymes.

**Table 5:** Inhibitory effects of test drugs (75 µM) on the enzymatic activity of recombinant AChE expressed in HEK 293 cells, and IC_50_ values for the inhibitory effects of test drugs (new compounds and reference inhibitors) on the enzymatic activity of human recombinant MAO isoforms expressed in baculovirus infected BTI insect cells.

Compound	AChE(µM)^a^	MAO-A^b^(IC_50_, µM )	MAO-B^b^(IC_50_, µM )
**Rasagiline**	82.5±4.3	ND	ND
**Clorgyline**	ND	0.0040±0.00025^c^	63.41±1.2
**Moclobemide**	ND	361.38±19.4^c^	>1000
**OXO 1**	26.2±5.6	27.32±1.18^c^	>100
**OXO 13**	25.6±5.9	2.12±0.07^c^	>50

All IC_50_ values shown are the means ± SEM from five experiments.

a Percentage inhibition.

b Values for clorgyline, moclobemide, OXO 1 and OXO 13 were taken from Prado-Prado et al. (21).

c *p*<0.01 relative to the corresponding IC_50_ values obtained against MAO-B, as determined by ANOVA/Dunnett’s.

### Bioinformatic Analysis of Other Putative *Leishmania* Targets for Oxoisoaporphine Compounds

Taking into consideration that the in vitro inhibition assays with the MAO and AChE enzymes did not indicate any differences between OXO **1** and OXO **13** compounds, we carried out a predictive study of putative targets among LPs. As there are no previous reports of any predictive method for drug-target interactions developed specifically for LPs, we developed a new model. The best LDA model found was as follows:
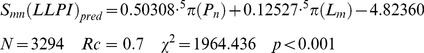
(2)where N is the number of cases (LLPIs and nLLPIs) used to train the model. This model has a Rc = 0.7 and an accuracy of 95.2% in training and of 94.8% in external validation series (Table 6). These results indicate the development of an accurate model according to previous reports on the use of LDA in drug discovery [25]. Nonetheless, the LDA model is unbalanced because only correctly classified ≈ 70% of LLPIs and 98% of nLLPIS. Therefore, the Artificial Neural Network (ANN) module of ST was used to search for a non-linear model [26]. The best ANN model was a Multi Layer Perceptron with 3 layers of neurons (MLP3). The MLP3 classifier presented an overall Ac = 92.5% in both training and external validation series (Table 6). In contrast to the LDA model, the ANN model is not unbalanced and correctly classified ≈ 92% of both LLPIs and nLLPIs. In addition, the MLP3 model is highly accurate, as demonstrated by the Receiver Operating Curve analysis, with an area under curve of 0.95. Finally, both LDA and MLP3 were used to predict the possible LLPIs between the 624 LPs and the 18 compounds. The predicted s_ij_ scores for LLPIs are sensitivity-weighted averages of the probabilities with which the compound i^th^ interact with protein j^th^ as determined by both the first linear method l1 = LDA and the first non-linear method nl1 = MLP3. The values were calculated as follows: *s(LLPI)_ij_* = ½·[Sn_l1_·p_nm_ *(LLPI)_ll_*+Sn_2_·p_nm_ *(LLPI)_nll_*], where Sn_l1_ and Sn_nl1_ are the sensitivities in training of methods m_l1_ and m_2_ respectively. This takes into account the predictions of the best two models found in proportion to their sensitivities (Table 6). All these proteins are predicted by both LDA & MLP3 to undergo LLPIs with all oxoisoaporphine derivatives with average sensitivity-weighted probabilities >0.80. Both models predicted as potential targets four proteins present in members of the Trypanosomatidae family, which were two putative pteridine reductase 2 (PTR2) proteins described in *Trypanosoma cruzi* (1MXF and 1MXH), one *N-*myristoyltransferase (NMT) from *L. donovani* (2WUU), and one topoisomerase I from *L. donovani* (2B9S).

**Table 6:** Results of LDA and ANN classification models.

Model		Data	Train				Validation		
Effects	Profile	Sub-set	nLLPIs	LLPIs	%	Stat.^a^	%	nLLPIs	LLPIs
Linear	LDA 1	nLLPIs	2,826	45	*98.4*	Sp_l1_	*98.0*	933	19
	2∶2-1∶1	LLPIs	113	310	*73.3*	Sn_l1_	*72.9*	38	102
		Total			*95.2*	Ac_l1_	*94.8*		
Non-Linear	MLP	nLLPIs	2,657	214	*92.5*	Sp_nl1_	*92.6*	882	70
	14∶14-9-1∶1	LLPIs	32	391	*92.4*	S_nl1_	*91.4*	12	128
		Total			*92.5*	Ac_nl1_	*92.5*		

a Stat. are the statistical parameters of the models in both training and validation series: Sp, Sn, and Ac indicate Specificity, Sensitivity and Accuracy. In addition, the subscripts l and nl indicate whether the models are linear or non-linear and the number in the subscripts indicates the number of the model, so that: Sp_l1_, Sn_l1_, Ac_l1_, and Spn_l1_, Sn_nl1_, Ac_nl1_, are the Specificities, Sensitivities, Accuracies of the first linear and non-linear models.

## Discussion

**Figure 3:**
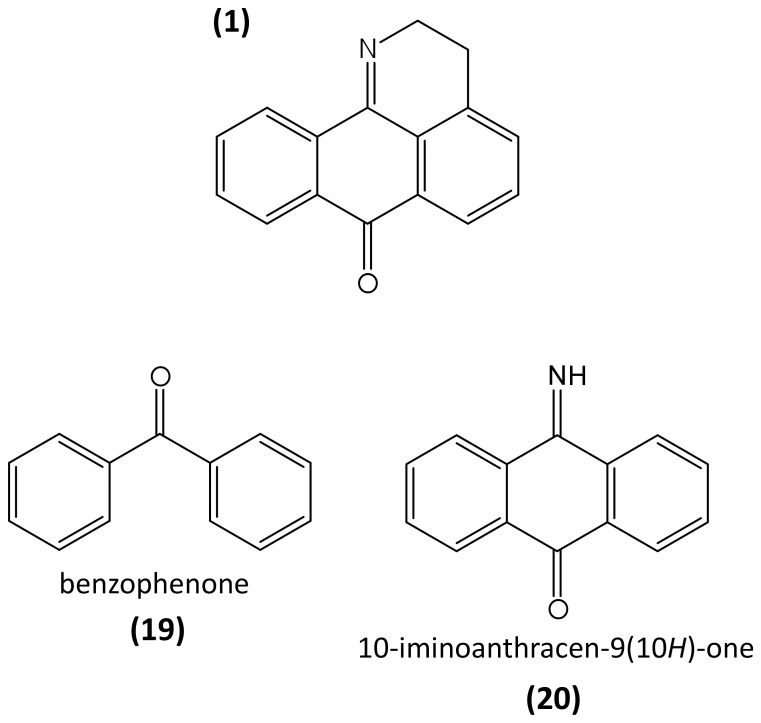
Structural comparison between benzophenone (19) and iminoanthraquinone (20) and the oxoisoaporphine scaffold (1).

In this study, we present data on novel oxoisoaporphines, some of which display either in vitro or in vivo antileishmanial activity. However, because only a limited number of compounds were active at low doses, the structural differences possibly related to the activity of this type of alkaloids are also discussed. Thus, the carbonyl group at C-7 and the 1,2-dihydro-isoquinoline system are common moieties in the active compounds OXO **1** and OXO **13**, which may be relevant to the observed antileishmanial activity. Nevertheless, in the D ring of compound OXO **13,** partial hydrogenation of the aromatic ring yields a cyclohexene that is not flat, so that this compound will interact differently from OXO **1**. The in vivo assay data with the OXO **1** and OXO **13** compounds showed a different ability to inhibit the growth of *Leishmania* amastigotes both in liver and in spleen of BALB/c mice. Thus, while OXO **1** showed a reduction in the parasite burden of almost 100% in liver and 78% in the spleen, OXO **13** did not show a significant inhibitory activity (Table 4). This indicates that the conservation of the iminoanthraquinone unit (**20**) in OXO **1** (Figure 3) may promote the leishmanicidal activity or a better bioavailability in the site of action than OXO **13**.

Regarding possible target(s) for the antileishmanial activity of oxoisoaporphine compounds, it has been reported that this family of molecules is able to inhibit the MAO-A enzyme selectively [21]. However, as MAO-A has not been reported to be present in Trypanosomatids, and the inhibitory activity of these compounds against MAO-A was the inverse of their antileishmanial activity, this enzyme can probably be rejected as a target for antileishmanial activity.

In addition to MAOs, acetyl/butyryl cholinesterases have also been reported to be inhibited by oxoisoaporphines and oxoaporphines [29]. Unlike for MAO-A, an AChE has been reported to be present in the glycosome of *T. evansi*, which might act by regulating the flow of Ca^+2^ between the organelle and the cytosol [30]. However, as with MAO-A, the present results (Table 5) showed that compounds OXO **1** and OXO **13** exerted only moderate inhibitory activity against AChE in comparison with the rasagiline used as a control, and more importantly, both compounds exerted similar inhibitory activity. Therefore, as with MAO-A, the *Leishmania* AChE also appears a highly unlikely target for the oxoisoaporphine compounds.

A bioinformatics model was developed to search the *Leishmania* proteome for alternative targets for the oxoisoaporphine compounds. Although a bioinformatics model based on neural networks cannot be expected to provide information about target proteins that could be selectively targeted by structurally related drugs, the proposed model may prove useful as a general model to identify proteins that merit investigation as targets for such compounds. As described in the above section, the model identified four proteins as highly likely putative targets, including two putative PTR2 (1MXF and 1MXH), one NMT (2WUU) and one topoisomerase I (2B9S).

Regarding 1MXF and 1MXH, reduced pteridines are known to be required for a number of important cellular functions. Unlike their mammalian hosts, trypanosomatid parasites are pteridine auxotrophs, and they salvage the precursor pteridines from the host to reduce them to the respective biologically active tetrahydro forms. In *Leishmania*, pteridine reductase 1 (PTR1), the primary enzyme for reducing pterins, is also responsible for resistance to antifolate drugs. Typically, PTR1 is more active with fully oxidized biopterin and folate than with their reduced counterparts. Other authors have identified an enzyme, TcPTR2 from *T. cruzi*, which, although very similar to PTR1 in its primary sequence, can only reduce dihydrobiopterin and dihydrofolate but not oxidized pteridines [31].

The NMT (2WUU) catalyses the attachment of myristate to the amino-terminal glycine residue of a subset of eukaryotic proteins that function in multiple cellular processes, including vesicular protein trafficking and signal transduction. *N-*myristoylation facilitates association of substrate proteins with membranes or hydrophobic domains of other partner peptides, which is essential for viability in all cell types tested to date. Previous studies have validated *L. donovani* NMT as a potential target for the development of new therapeutic agents against visceral leishmaniasis [32].

Finally, the type I topoisomerases, such as 2B9S, are essential enzymes that are responsible for relaxing superhelical tension in DNA [33]. Interestingly, topoisomerase I is a target for anti-cancer drugs such as camptothecin [34]. In addition, topoisomerase II has been reported to be inhibited by oxoaporphines (e.g. dicentrinone) that display potent inhibitory activity against *L. braziliensis* and *T. cruzi* [10].

In summary, we investigated for the first time the in vitro and in vivo activity of a group of novel oxoisoaporphine compounds, some of which display inhibitory activity against several *Leishmania* species. We have also developed a bioinformatics tool that may be useful for searching for new targets for antileishmanial drugs.
